# High-resolution 3T-MRI with microcoil enhancement for preoperative evaluation of cutaneous squamous cell carcinoma: a case report and literature review

**DOI:** 10.3389/fmed.2026.1686516

**Published:** 2026-01-23

**Authors:** Shun Cao, Shaowei Zhan, Mengxiao Tang, Qiuyu Yu, Hongjie Hu

**Affiliations:** Department of Radiology, Sir Run Run Shaw Hospital, Zhejiang University School of Medicine, Hangzhou, China

**Keywords:** 3T-magnetic resonance imaging (3T-MRI), cutaneous squamous cell carcinoma (cSCC), cutaneous tumors, high-resolution magnetic resonance imaging (HR-MRI), magnetic resonance imaging (MRI), microcoil, preoperative assessment, tumor infiltration depth

## Abstract

**Background:**

Cutaneous squamous cell carcinoma (cSCC) is a common non-melanoma skin cancer with potential for local invasion and metastasis. Accurate preoperative assessment is essential for optimal treatment planning.

**Materials and methods:**

We report a case of an 83-year-old female patient who presented with a progressively enlarging scalp mass over 3 months. HR-MRI revealed a mixed-signal lesion (22 × 15 × 26 mm) in the right scalp. On T1-weighted imaging (T1WI) and T2-weighted imaging (T2WI), the lesion exhibited heterogeneous signals with an irregular, crater-like surface. Post-contrast imaging demonstrated significant heterogeneous enhancement. The tumor was located within the epidermis, dermis, and subcutaneous fat, closely adhering to the galea aponeurotica with well-defined margins. Surgical resection and histopathological examination confirmed a (exophytic type) moderately to well-differentiated cSCC (2.8 × 2.3 × 2.0 cm) infiltrating the subcutaneous tissue but without perineural invasion or deeper tissue involvement.

**Results:**

HR-MRI provided clear visualization of tumor morphology, infiltration depth, and relationship with surrounding structures. Compared to conventional MRI, HR-MRI improved the accuracy of tumor boundary delineation, offering valuable information for preoperative planning.

**Conclusion:**

HR-MRI plays a significant role in the evaluation of cSCC, particularly in assessing tumor infiltration depth and differentiating it from other cutaneous malignancies. Its high-resolution imaging facilitates early detection, precise surgical planning, and improved patient outcomes.

## Introduction

Cutaneous squamous cell carcinoma (cSCC) is the second most common non-melanoma skin cancer (NMSC) following basal cell carcinoma(BCC) (1). The incidence of skin cancer is rising, particularly in older populations, with cSCC being a leading cause of skin cancer-related morbidity. Although surgical excision is typically effective, some cSCC cases are associated with a higher risk of recurrence, metastasis, and mortality (2). Clinically, cSCC often presents as a rapidly enlarging mass or a non-healing ulcer. Diagnosis is based on histopathology, with wide local excision being the most commonly used treatment. However, assessing the exact tumor size and depth of infiltration remains challenging and is often based on clinical judgment or dermoscopy, which can lead to suboptimal prognostic outcomes and delayed recovery (3–5). Magnetic resonance imaging (MRI) is a valuable tool for assessing cSCC, particularly in larger tumors, by providing detailed information on tumor invasion and its relationship to surrounding structures (6, 7). However, conventional MRI techniques can be limited in accurately delineating tumor margins, particularly in smaller or superficially invasive tumors. High-resolution 3T-MRI with Microcoil Enhancement offers improved resolution, allowing for better visualization of early invasion and microstructural changes, potentially enhancing diagnostic accuracy.

This study aims to demonstrate the clinical utility of microcoil-enhanced 3T-MRI in the evaluation of cutaneous squamous cell carcinoma (cSCC), using a pathologically confirmed case of scalp cSCC. Unlike previous studies that primarily addressed the technical feasibility of high-resolution MRI for cutaneous tumors, this case emphasizes the incremental preoperative value of microcoil-enhanced 3T-MRI over conventional MRI, particularly in the accurate delineation of tumor margins and assessment of infiltration depth, which are critical for surgical planning.

## Case report

### Clinical data

An 83-year-old female patient presented with a progressively enlarging scalp mass over 3 months. Initially asymptomatic, the lesion was later evaluated due to its increasing size.

### Imaging assessment

A 3T-MRI scanner (VIDA, Siemens Medical Solutions, Erlangen, Germany) with a high-resolution microscopy coil was used. T2-weighted fast spin-echo (FSE) imaging was performed in the coronal plane with parameters: TR/TE 2447/100 ms, slice thickness 2 mm, field of view (FOV) 70 × 70 mm, matrix 256 × 256, and echo train length (ETL) 12. T1-weighted FSE imaging was also performed in the coronal plane with TR/TE 400/22 ms, maintaining the same slice thickness, FOV, and matrix as T2WI, with an ETL of 4. Post-contrast T1-weighted FSE imaging was performed after intravenous administration of 0.1 mmol/kg contrast agent.

**Figure 1:**
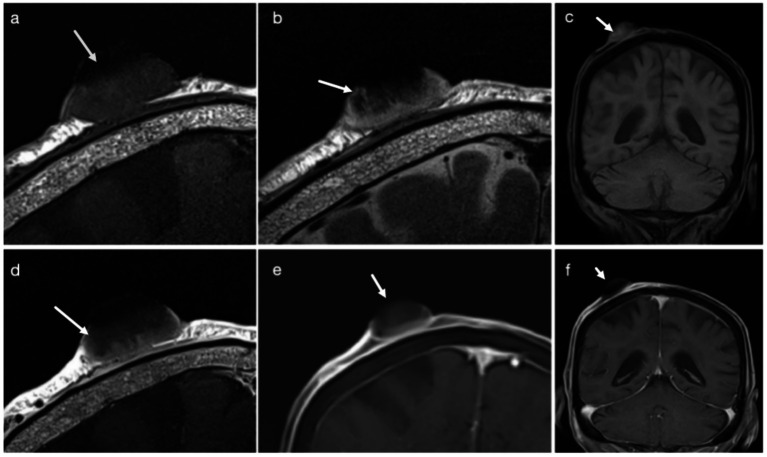
3T-MRI findings in a case of squamous cell carcinoma in an 83-year-old female. **(a)** Coronal T1-weighted 3T-MRI demonstrates an oval-shaped lesion with heterogeneous hypointense signals and an irregular surface contour (arrow). **(b)** Coronal T2-weighted 3T-MRI reveals a mixed hyperintense mass with superficial ulceration (arrow) and adjacent linear hyperintense foci, indicative of peritumoral inflammatory changes. **(c)** Conventional T1-weighted fat-suppressed MRI shows ill-defined lesion margins (arrow). **(d)** Post-contrast coronal T1-weighted MRI highlights heterogeneous enhancement (arrow), correlating with tumor angiogenesis. **(e)** T1-weighted fat-suppressed MRI sequence accurately delineates the involvement of the dermal-subcutaneous junction (arrow). **(f)** Conventional T1-weighted fat-suppressed imaging exhibits reduced soft tissue contrast resolution compared to MRI (arrow).

HR-MRI revealed a mixed-signal lesion in the right scalp. The lesion demonstrated heterogeneous signal intensity on both T1-weighted and T2-weighted images, with intermixed hypo- to isointense components on T1WI and predominantly hyperintense areas on T2WI, likely reflecting keratinization and associated inflammatory components. The lesion exhibited a well-defined base and close adherence to the galea aponeurotica. On microcoil-enhanced HR-MRI, the lesion measured approximately 22 × 15 × 26 mm along the maximum axial and craniocaudal dimensions. Protruding from the scalp surface with an irregular, crater-like appearance. Post-contrast imaging demonstrated significant heterogeneous enhancement (Figure 1). Minor discrepancies between MRI-based measurements and histopathological dimensions may be partially attributed to differences in measurement orientation and tissue shrinkage following formalin fixation. Surgical resection was performed the following day.

**Figure 2:**
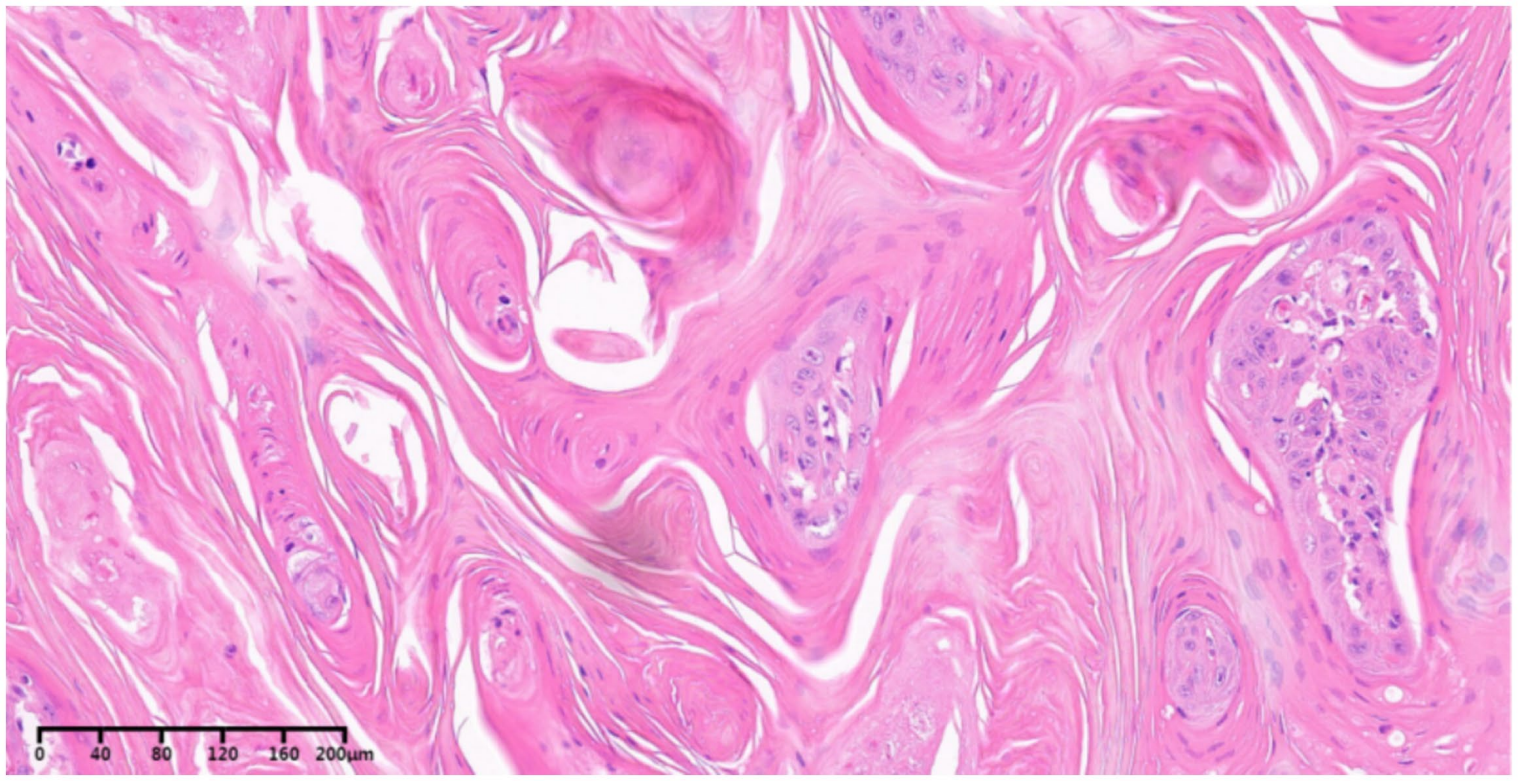
Histological specimen (hematoxylin and eosin staining) reveals tumor cells of cutaneous squamous cell carcinoma arranged in a trabecular pattern, with abundant keratin pearls present. The tumor cells exhibit large size, abundant eosinophilic cytoplasm, and large, vacuolated nuclei with prominent nucleoli. The surrounding stromal components are distinctly evident.

Histopathological findings: Histopathological examination confirmed an exophytic, moderately to well-differentiated cSCC measuring 2.8 × 2.3 × 2.0 cm, with a height of 2.0 cm (Figure 2). The tumor infiltrated the subcutaneous tissue but had negative margins, with no evidence of perineural invasion or deeper tissue involvement.

## Discussion

Although uncomplicated cSCC can often be cured with surgery combined with local therapy, there remains a clear clinical need for more objective and noninvasive diagnostic tools to guide clinicians in developing detailed biopsy strategies and surgical plans for lesions with atypical features or suspected subcutaneous extension (8). Commonly used modalities—such as high-frequency ultrasound (HFUS) (9), optical coherence tomography (OCT) (10), dermoscopy (11), and CT (12)—provide useful structural information but have notable limitations. HFUS offers excellent axial resolution but limited penetration, reducing its ability to assess deeper margins. OCT provides near-histologic visualization but is restricted to a depth of 1–2 mm. Dermoscopy is confined to surface morphology, and CT lacks sufficient soft-tissue contrast for thin cutaneous lesions (9–12). In contrast, HR-MRI combines high spatial resolution with adequate tissue penetration, enabling three-dimensional assessment of tumor thickness, peritumoral inflammation, and involvement of deeper structures. Thus, HR-MRI provides more comprehensive preoperative information than HFUS or OCT, especially for lesions in which deeper invasion must be excluded.

High-resolution MRI is a noninvasive technique capable of quasi-histologic, *in vivo* visualization of the skin (13). By employing small surface coils (<100 mm) in combination with a 3T or higher MRI system, HR-MRI achieves substantially higher spatial resolution than conventional surface coils (13). This capability allows for visualization of tissue structures not identifiable with standard imaging techniques. Its primary clinical value lies in delineating deep and peripheral tumor margins, providing dermatologic surgeons—particularly those performing Mohs micrographic surgery—with a reliable preoperative roadmap (14). HR-MRI has also been successfully applied to imaging superficial anatomical structures such as the orbit (15), parotid gland (16), and small joints of the hands and feet (17, 18).

Although high-resolution or microcoil-based MRI has been previously reported in the evaluation of various cutaneous tumors, its direct contribution to preoperative decision-making in cutaneous squamous cell carcinoma (cSCC) remains under-reported. This case illustrates how microcoil-enhanced high-resolution MRI can provide clinically actionable information beyond conventional MRI, particularly for surgical planning. In this case of scalp cutaneous squamous cell carcinoma (cSCC), 3T-MRI with Microcoil Enhancement demonstrated significant value by providing detailed visualization of tumor morphology, infiltration depth, and its relationship with surrounding tissues, thereby improving the accuracy of preoperative assessment. The tumor displayed typical exophytic features of cSCC, including an ovoid or cauliflower-like appearance with ulceration and a crater-like surface. These findings are consistent with previous reports, where MRI has proven effective in identifying characteristic tumor features and assessing their extent (19).

A major strength of this examination was the superior performance of microcoil-enhanced HR-MRI compared with conventional MRI. Three critical advantages were observed. First, the use of a microcoil markedly increased signal-to-noise ratio (SNR) by reducing coil–tissue distance, enabling thinner slices and minimizing partial-volume effects. This enhancement allowed visualization of microstructural features—such as dermal–subcutaneous junction disruption and crater-like ulceration—that are often indistinct on routine MRI. Second, HR-MRI improved staging accuracy by differentiating tumor-confined involvement of the fat layer from deeper galeal or fascial invasion. Third, the technique facilitated preoperative planning by enabling precise delineation of tumor margins; in this case, an approximate 2-mm boundary was identified and later confirmed histologically.

The results of this case further support the potential of HR-MRI in differentiating cSCC from other cutaneous malignancies, including basal cell carcinoma (BCC), melanoma, and cutaneous lymphoma. While these findings align with existing literature, they also highlight the superior resolution and enhanced image contrast provided by HR-MRI compared with conventional MRI, especially for tumors with irregular borders or superficial invasions (Table 1).

**Table 1:** Differential diagnosis of cutaneous squamous cell carcinoma in the head and neck region (20).

Differential diagnosis	Typical locations	Clinical features	MRI characteristics	Other features
Basal cell carcinoma (BCC)	Face (e.g., periorbital, nasal areas)	Usually non-ulcerated, slow-growing	Relatively homogeneous low signal intensity	Locally invasive, rarely metastasizes
Cutaneous malignant melanoma	Extremities, back, head and neck	Pigmented lesions or nodules, rapid progression	Heterogeneous enhancement, sometimes high signal on T1WI	Often associated with regional lymphadenopathy
Cutaneous ulcer	Sites prone to trauma or underlying pathology	Surface ulceration due to trauma, diabetes, vascular disease	relatively homogeneous signal; often involves subcutaneous fat; minimal to moderate enhancement.	Clear history, non-tumorous behavior
Keratoacanthoma	Sun-exposed areas (face, hands)	Volcano-like nodules with central depression, self-limiting	clear boundaries; sometimes slightly elevated above the skin surface; irregular skin contour; mild enhancement.	Spontaneous resolution within months, minimal metastasis
Cutaneous lymphoma	Limbs, trunk, head and face	Red plaques or nodules, often with itching	Diffuse skin thickening or nodular high signal	T-cell origin; immunohistochemistry CD3/CD4 positive

### Limitations

Despite these advantages, there are limitations to this study. The case report design restricts generalizability, and the findings are based on a single patient’s data. Additionally, while 3T-MRI provided detailed information on tumor boundaries, it remains subject to limitations inherent to MRI, such as motion artifacts and reliance on high-quality imaging equipment. Further studies with larger cohorts and prospective designs are necessary to validate the broader applicability of 3T-MRI in cSCC and other skin cancers.

## Conclusion

This case suggests that microcoil-enhanced 3T high-resolution MRI may offer incremental value over conventional MRI in the preoperative assessment of cutaneous squamous cell carcinoma. Although further studies with larger cohorts are required to validate these findings, HR-MRI appears to be a promising tool for improving preoperative evaluation and surgical planning in cSCC.

## Data Availability

The datasets presented in this study can be found in online repositories. The names of the repository/repositories and accession number(s) can be found in the article/supplementary material.
